# RareLink: scalable REDCap-based framework for rare disease interoperability linking international registries to FHIR and Phenopackets

**DOI:** 10.1038/s41525-025-00534-z

**Published:** 2025-11-18

**Authors:** Adam S. L. Graefe, Filip Rehburg, Samer Alkarkoukly, Daniel Danis, Ana Grönke, Miriam R. Hübner, Alexander Bartschke, Thomas Debertshäuser, Sophie A. I. Klopfenstein, Julian Saß, Julia Fleck, Mirko Rehberg, Jana Zschüntzsch, Elisabeth F. Nyoungui, Tatiana Kalashnikova, Luis Murguía-Favela, Beata Derfalvi, Nicola A. M. Wright, Shahida Moosa, Soichi Ogishima, Oliver Semler, Susanna Wiegand, Peter Kühnen, Christopher J. Mungall, Melissa A. Haendel, Peter N. Robinson, Sylvia Thun, Oya Beyan

**Affiliations:** 1 Berlin Institute of Health at Charité‒Universitätsmedizin Berlin, Berlin, Germany; 2 Institute for Biomedical Informatics, University of Cologne, Faculty of Medicine and University Hospital Cologne, Cologne, Germany; 3 Medical Data Integration Center (MeDIC), University of Cologne, Faculty of Medicine and University Hospital Cologne, Cologne, Germany; 4 Center for Rare Diseases, Medical Faculty, RWTH Aachen University, Aachen, Germany; 5 Department of Pediatrics and Adolescent Medicine, Faculty of Medicine and University Hospital Cologne University of Cologne Cologne Germany, Cologne, Germany; 6 Department of Neurology, University Medical Center Goettingen, Goettingen, Germany; 7 Department of Medical Informatics, University Medical Center Goettingen, Goettingen, Germany; 8 Section of Hematology and Immunology, Alberta Children’s Hospital, Calgary, Canada; 9 University of Calgary, Calgary, Canada; 10 Department of Pediatrics, Division of Immunology, Dalhousie University, IWK Health, Halifax, NS Canada; 11 Division of Molecular Biology and Human Genetics, Stellenbosch University, Cape Town, South Africa; 12 Medical Genetics, Tygerberg Hospital, Cape Town, South Africa; 13 INGEM, Tohoku University, Sendai, Miyagi Japan; 14 Department of Informatics for Genomic Medicine, ToMMo, Tohoku University, Sendai, Miyagi Japan; 15 Department of Pediatric Endocrinology and Diabetology, Charité University Hospital, Berlin, Germany; 16 Center for Chronically Sick Children, Charité Universitätsmedizin Berlin, Berlin, Germany; 17 Berlin Center for Rare Diseases—Charité University Hospital, Berlin, Germany; 18 Deutsches Zentrum für Kinder- und Jugendgesundheit (DZKJ), partner site Berlin, Berlin, Germany; 19 Division of Environmental Genomics and Systems Biology, Lawrence Berkeley National Laboratory, Berkeley, CA USA; 20 Department of Genetics, University of North Carolina Chapel Hill, Chapel Hill, NC USA; 21 The Jackson Laboratory for Genomic Medicine, Farmington, CT USA; 22 Fraunhofer Institute for Applied Information Technology, Sankt Augustin, Germany

**Keywords:** Endocrine system and metabolic diseases, Immunological disorders, Neurological disorders, Genetics research, Paediatric research, Translational research, Paediatrics, Diagnosis, Data mining, Data processing, Databases, Gene ontology, Genome informatics, Functional genomics, Genomics

## Abstract

While Research Electronic Data Capture (REDCap) is widely adopted in rare disease research, its unconstrained data format often lacks native interoperability with global health standards, limiting secondary use. We developed *RareLink*, an open-source framework implementing our published ontology-based rare disease common data model. It enables standardised data exchange between REDCap, international registries, and downstream analysis tools by linking Global Alliance for Genomics and Health Phenopackets and Health Level 7 Fast Healthcare Interoperability Resources (FHIR) instances conforming to International Patient Summary and Genomics Reporting profiles. RareLink was developed in three phases across Germany, Canada, South Africa, and Japan for registry and data analysis purposes. We defined a simulated Kabuki syndrome cohort and demonstrated data export to Phenopackets and FHIR. RareLink can enhance the clinical utility of REDCap through its global applicability, supporting equitable rare disease research. Broader adoption and coordination with international entities are thus essential to realise its full potential.

## Introduction

Rare diseases represent a significant global health challenge with over 260 million affected worldwide. In Europe, a condition is classified as rare when it affects fewer than 5 of 10,000 people^1^. More than 10,000 distinct rare diseases have been identified^2^, the majority of which are genetic in origin and first manifest during childhood^1^. Diagnostic delays are common, often spanning several years^3^, and most rare diseases currently lack causal treatments^4^. Despite their collective impact, the field continues to face substantial barriers, including limited data quality and availability, complicating diagnosis, optimal care, and research progress. Limited resources and heterogeneous, non-interoperable information systems compound these challenges, resulting in rare disease datasets not conforming to international data standards^5^.

Due to the inherent fragmentation of rare disease individuals across institutions and health systems, interoperability is essential to enable consistent data interpretation and exchange for secondary use supporting research on rare diseases^6^. Adoption of medical ontologies, terminologies, Health Level 7 (HL7) Fast Healthcare Interoperability Resources (FHIR), and the Global Alliance for Genomics and Health (GA4GH) Phenopacket Schema designed by the Monarch Initiative enables reliable data exchange, supporting registries, secondary data use, and precision medicine^6–9^. These standards support many of the FAIR guiding principles—making data more *findable*, *accessible*, *interoperable*, and *reusable* across distributed systems—thereby supporting scalable, collaborative rare disease research^10^.

However, many hospital information systems neither include native rare disease–specific data elements nor natively support the integration of the required terminology and data standards, limiting both systematic data collection and precise encoding, and thereby posing barriers to clinical and translational use^11^. Research Electronic Data Capture (REDCap) is a globally adopted, web-based, no-cost software for non-profit organisations frequently used for data collection in research^12^ and rare disease registries^5^. Importantly, all data is stored on-site within the hosting institution’s servers, ensuring data sovereignty and compliance with applicable data protection regulations. While its flexibility in project and data schema definitions allows simple usage, it often results in unconstrained, project-specific data schemas that restrict interoperability, complicating alignment with international data standards, cross-registry data sharing, and federated downstream analyses^13^.

In our previous work, we developed an ontology-based rare disease common data model (RD-CDM) harmonising the European Rare Disease Registry Infrastructure Common Data Set (ERDRI-CDS), FHIR, and Phenopackets^14^. Building on this foundation, we now present *RareLink*, a novel REDCap-based framework that integrates this model by predefining data collection instruments with embedded ontology codes. RareLink is configured to enable automated export to FHIR resource instances and Phenopackets via a command-line-interface, supported by documentation that guides users through each step of the workflow. This study presents the iterative development and evaluation of RareLink along with the generated documentation and software across real-world settings in Germany, Canada, South Africa, and Japan, covering diverse use cases and a broad spectrum of rare diseases.

## Results

**Fig. 1:**
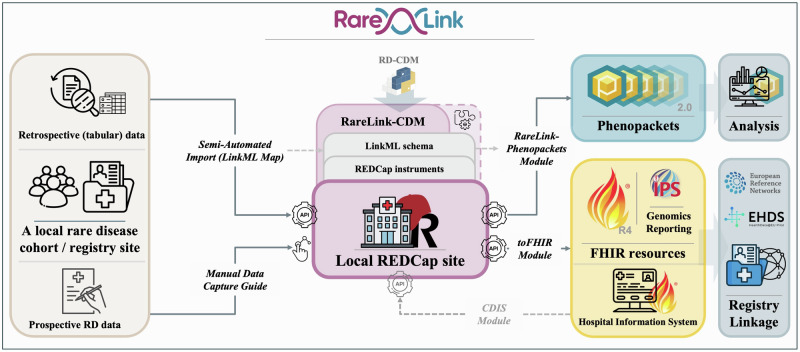
Overview of the entire RareLink framework’s data flow. The RareLink framework is integrated with a local REDCap instance and preconfigured for the RareLink-CDM, with the option of disease-specific extensions. The RareLink-CDM is equivalent to the ontology-based RD-CDM integrated through its Python package and defined through its LinkML schema and the corresponding REDCap instruments. Utilising the local REDCap API and the RareLink-CLI, the toFHIR & CDIS modules can export to HL7 FHIR International Patient Summary (IPS) and Genomic Reporting resources and import from a corresponding FHIR server enabling export or record linkage to the European Reference Networks, the EHDS, or other international and domain-specific registries. The RareLink-Phenopackets module exports to GA4GH Phenopackets, allowing for the use of Phenopacket-based analysis software. Additionally, the LinkML-based import mapper can support data import from tabular databases, map it to the corresponding LinkML schema, and enable either subsequent import into REDCap or direct export as Phenopackets. The Manual Data Capture Guide aids with the manual entry of data according to the common data model. API Application Programming Interface. CDIS Clinical Data Interoperability Services. CDM Common Data Model. CLI Command Line Interface. EHDS European Health Data Space. FHIR Fast Healthcare Interoperability Resources. GA4GH Global Alliance for Genomics and Health. IPS International Patient Summary. LinkML Linked Data Modeling Language. RareLink documentation https://rarelink.readthedocs.io/en/latest/index.html. RD-CDM Pyton Package Index (PyPI) https://pypi.org/project/rd-cdm/. RD-CDM ontology-based rare disease common data model (https://github.com/BIH-CEI/rd-cdm). REDCap Research Electronic Data Capture.

The RareLink framework is an open-source, modular software designed for seamless integration with any REDCap instance that is connected to the BioPortal Ontology Services terminology server and has a valid country-specific Systemized Nomenclature of Medicine—Clinical Terms (SNOMED CT) license (Fig. 1). RareLink consists of five core modules: (i) RareLink Documentation, (ii) RareLink Common Data Model, (iii) RareLink Command-Line Interface (CLI), (iv) RareLink FHIR Module, and (v) RareLink-Phenopackets Module. It is compatible with local REDCap projects and institutional deployments, supporting diverse use cases, including patient registries, study feasibility and cohort generation, and related clinical research activities. While RareLink can be deployed locally and core functions such as the FHIR export pipeline and the semi-automated import operate offline, an internet connection is required to access the documentation, support ontology services in the Phenopackets pipeline and term search in REDCap instruments. The framework is designed to be user-friendly for both clinical and technical personnel. By integrating the complete ontology-based RD-CDM^14^ within REDCap, RareLink provides a flexible framework that enables guided manual data entry for prospective cohorts and semi-automatic import using LinkML-Map^15^ for retrospective data sets. Once data is captured, users can export to validated HL7 FHIR instances and GA4GH Phenopackets by using the CLI that enables them to interact with the toFHIR Module and the RareLink-Phenopackets Module (Fig. 1). The Clinical Data Interoperability Services (CDIS) module^16^ can be integrated to import existing FHIR instances to REDCap. Although SNOMED CT and several of the other ontologies support multilingual labels, the current RareLink implementation retrieves ontology content from BioPortal using English-language labels only.

### RareLink documentation

The comprehensive RareLink documentation was designed with an emphasis on reusability, scalability, and cross-institutional applicability. Given RareLink’s deployment across multiple countries and institutions, clear and accessible documentation was essential to ensure consistent implementation and independent use. It centralises all RareLink features, guides, and resources, accessible from any REDCap site, and is structured into five subsections: Background, RareLink Framework, Installation, User Guide, and Additional Information. The *Background* section provides theoretical context and introduces the ontologies and terminologies employed—such as SNOMED CT—as well as the structural data standards such FHIR and Phenopackets, with references for further reading. The *RareLink Framework* section offers an overview of the architecture, including the RareLink-CDM and the command-line interface. *Installation* guides users through framework setup, REDCap configuration, data dictionary import, and REDCap API connection. Detailed user guides support manual data capture, semi-automated data entry, use of the Phenopackets and FHIR modules, development of REDCap instruments, and REDCap tools integration. The *Additional Information* section includes contribution guidelines, a changelog, FAQs, acknowledgements, licensing, and contact information (https://rarelink.readthedocs.io/en/latest/index.html).

### RareLink common data model

**Fig. 2:**
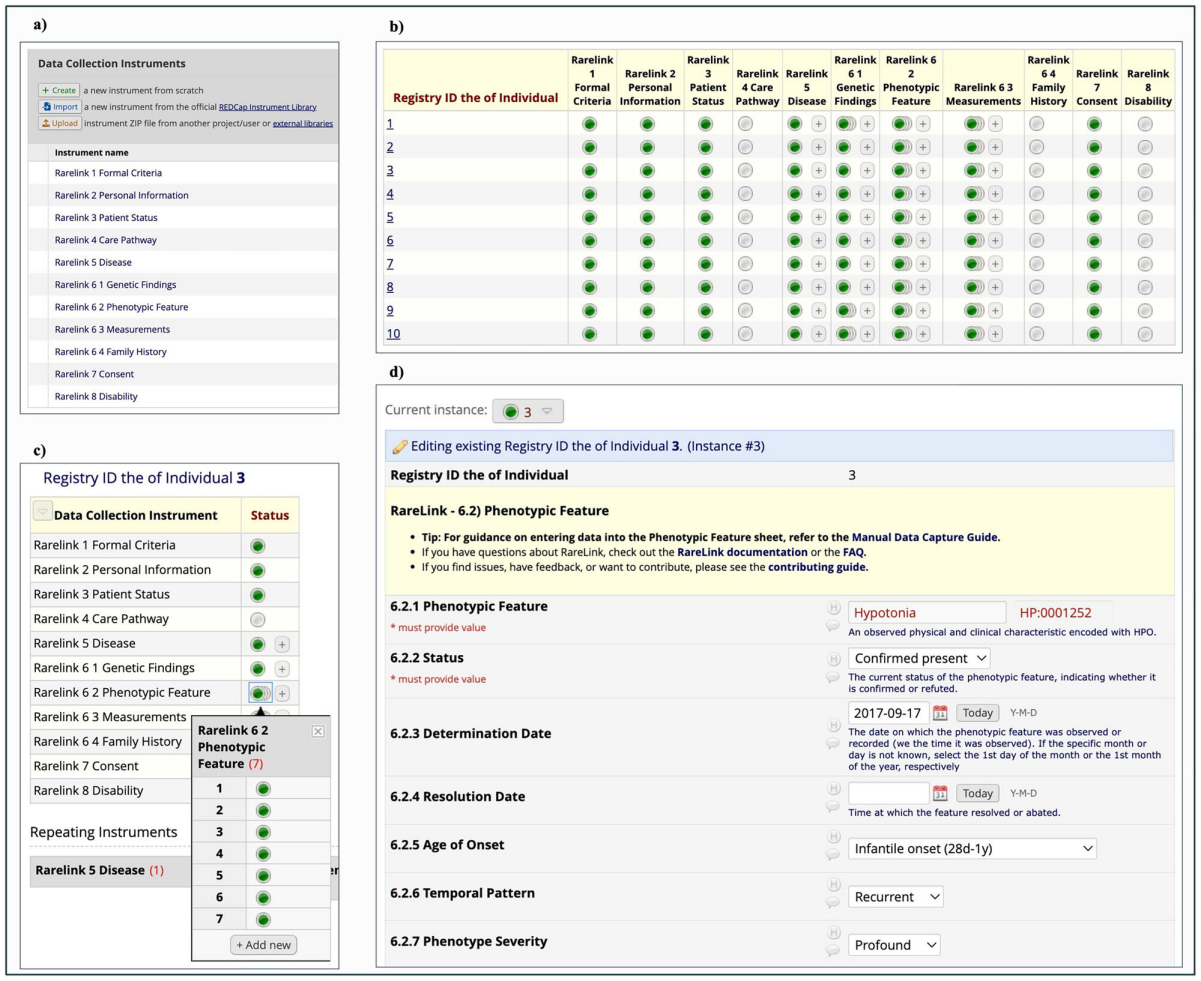
The RareLink-CDM in REDCap for the evaluation cohort of ten individuals with Kabuki syndrome type 1. **a** Overview of all RareLink-CDM sections displayed as standalone data collection instruments within REDCap’s Designer view. **b** The record status dashboard displaying the ten simulated individuals enrolled in the registry, each with completed data across demographic, consent, disease, genetic, phenotypic, and measurement instruments. **c** The record dashboard for individual 3, illustrating the use of repeating instances for phenotypic features. **d** A screenshot of the data entry window for a phenotypic feature of individual 3, including introductory text and links to the documentation and manual data capture guide. In this example, the individual was reported with confirmed, recurrent, and profound hypotonia, with an infantile onset dated 17 September 2017. HP Human Phenotype Ontology.

We enhanced the ontology-based RD-CDM^14^ for its REDCap implementation by developing the RareLink Common Data Model (RareLink-CDM), aligning it with corresponding FHIR resources and profiles, and Phenopacket blocks. To ensure interoperability, we defined required fields within each section and extended the Measurements section to support linkage with International Patient Summary (IPS) profiles for laboratory, imaging, and procedural data and Phenopacket’s MedicalAction block. Only the Formal Criteria section is mandatory; all other sections are optional but conditionally required: if any field is used, its associated mandatory elements must be completed. With exception of the Formal Criteria, Personal Information, Patient Status, Consent, and Disability, all sections are implemented as repeating instruments (Fig. 2a–c).

To promote accurate data capture and reduce entry errors, the REDCap instruments include detailed instructions, branching logic, and embedded links, complemented by the manual data capture guide (Fig. 2d). The LinkML representation of the RareLink-CDM mirrors this structure by grouping repeating instances in its JSON serialisation. Once data is exported as REDCap-JSON, it is automatically processed and validated against the LinkML-JSON schema. This schema^17^, along with the corresponding Python classes, includes all REDCap variables, coded terms, and value sets as importable enumerations. The RareLink-CDM automatically obtains its ontology versions from the RD-CDM Python package^18^, which in turn retrieves and validates the latest releases from BioPortal.

### Command line interface

**Fig. 3:**
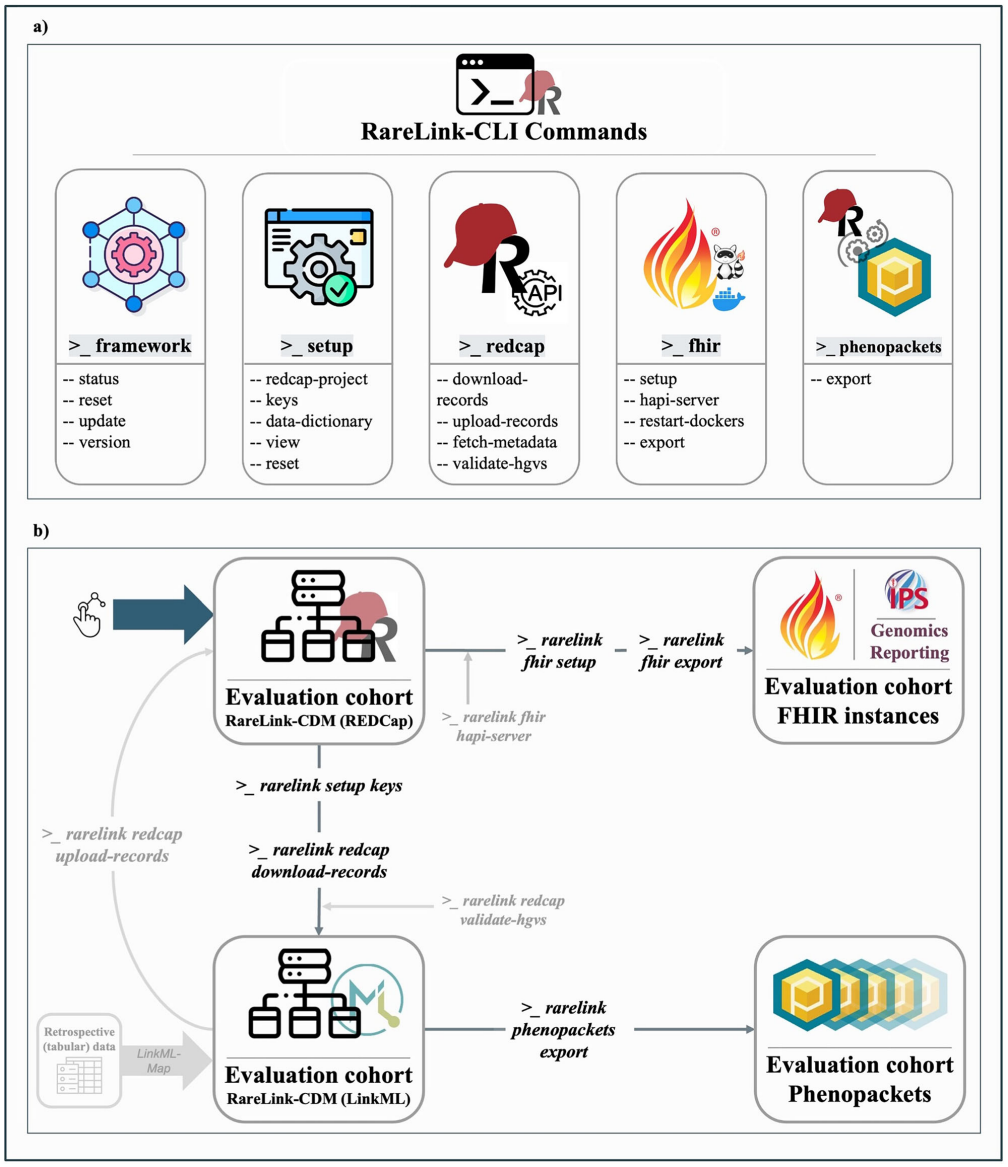
RareLink command-line interface and data flow for the Kabuki Type 1 evaluation cohort. **a** The RareLink CLI is organised into five primary command groups—*framework* (global configuration), *setup* (local installation), *redcap* (interaction with a REDCap project), *fhir* (FHIR export via the RareLink-FHIR module) and *phenopackets* (Phenopacket generation via the RareLink-Phenopackets module)—with further subcommands under each. Future releases will extend functionality as requirements evolve. **b** Key data-flow steps (*download-records*, *phenopackets export*, *fhir export*) are invoked after setup (*setup keys*, *fhir setup*, and, if necessary, *fhir hapi-server* and *redcap validate-hgvs)* on the CLI to fetch, validate, and transform REDCap data into the LinkML representation of the RareLink-CDM, Phenopackets, and FHIR instances (Supplementary Fig. 1 for full console output). The LinkML data can be imported back into REDCap using the *redcap upload-records* command. If the evaluation cohort had been imported from another retrospective database, LinkML-Map could also have been used. CDM common data model. FHIR Fast Healthcare Interoperability Resources. LinkML unified data modeling language. REDCap Research Electronic Data Capture.

The RareLink command-line interface (CLI) guides users through the entire workflow from setup to data export, with a design that accommodates those with limited coding experience. It features a user-friendly interface with descriptive commands, embedded links, and contextual hints. Each command includes cross-references and built-in validation to ensure required steps are completed and configurations are correct. Organised into five command groups, the CLI facilitates onboarding and streamline usage (Fig. 3a). After setup, the *redcap* command group enables API-based interaction with local REDCap projects. The *fhir* and *phenopackets* command groups provide all necessary steps for exporting data in both formats (Fig. 3b). As adoption of RareLink grows, additional commands will be incorporated based on community-driven feedback to support evolving needs.

### RareLink-FHIR-module

**Fig. 4:**
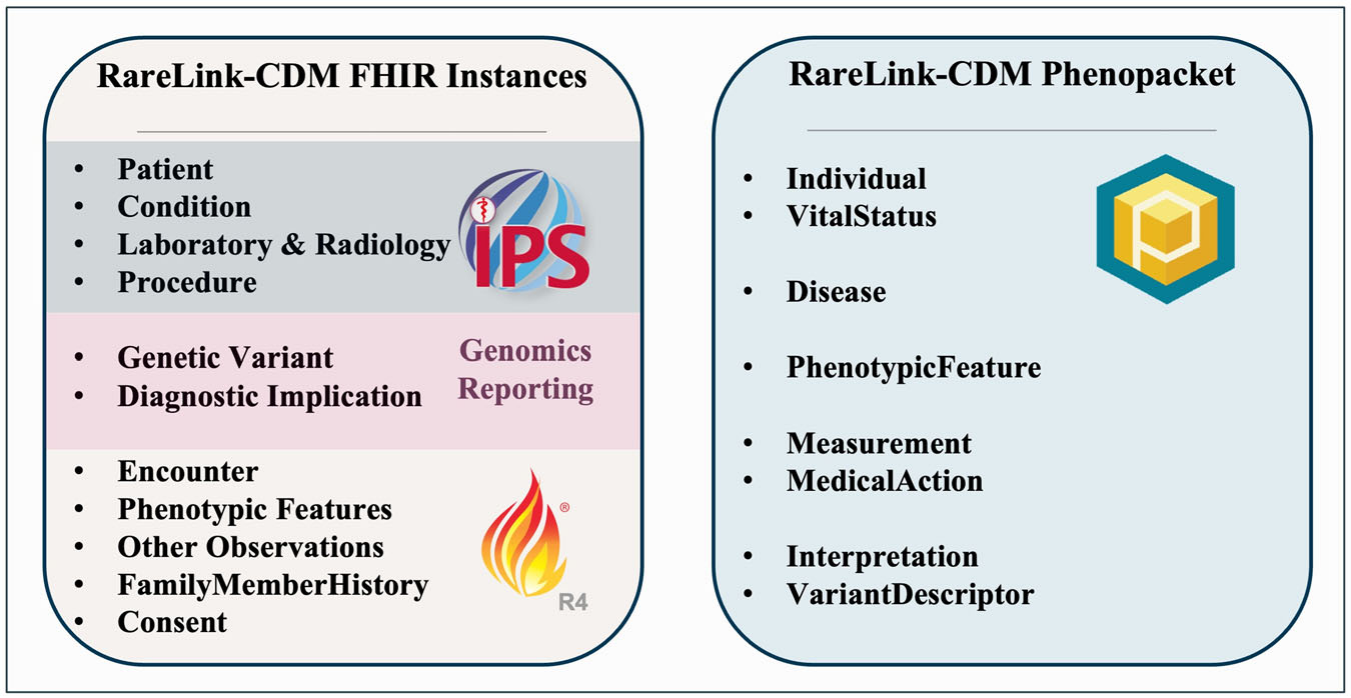
Schematic overview of the RareLink-CDM as both FHIR instances and a Phenopacket. FHIR instances conform to the HL7 International Patient Summary v2.0.0 profiles for Patient, Condition, Laboratory, Radiology, and Procedure. Genetic findings are captured using the HL7 Genomics Reporting v3.0.0 Genetic Variant and Diagnostic Implication profiles. Additional components, including encounters, phenotypic, and other observations (e.g. age category, gestational age), family history and consent (incorporating ERDRI-CDS elements), utilise FHIR R4 base resources. The RareLink-CDM Phenopacket comprises Individual, VitalStatus, and Disease blocks, together with phenotypic, measurement, and medical action data, and genetic information within the Interpretation and VariantDescriptor blocks. ERDRI-CDS European Rare Disease Infrastructure Common Data Set. HL7 Health Level 7. IPS International Patient Summary. RareLink-CDM RareLink Common Data Model.

All 75 elements mapped to FHIR in the ontology-based RD-CDM^14^ are exportable from the RareLink-CDM to the corresponding FHIR resources and profiles that we selected. The export process leverages the toFHIR engine^19^, which enables automatic validation against the IPS v2.0.0 profiles, the Genomics Reporting v3.0.0 profiles, and Base Resources v4.0.1 structure definitions to any FHIR server (Fig. 4). These profiles are embedded as dependencies within the RareLink-CDM profiles ensuring interoperability upon implementation. This functionality facilitates linkage to FHIR repositories, international registries, and supports data import via the Clinical Data Interoperability Services module^16^. The export pipeline requires Docker to be installed and running which can be hindered on some hospital systems by institutional software installation restrictions. In the absence of a remote FHIR server, a local instance can be set up using a HAPI server. Detailed guidance and all CLI commands are provided in the user guide documentation under the FHIR module section^17^. The RareLink-CDM FHIR implementation guide and specifications are publicly available and hosted through our repository^17^.

### RareLink-Phenopackets-module

With the exception of the family history section, all 43 elements mapped to Phenopackets in the ontology-based RD-CDM^14^ are exportable from REDCap via the RareLink-CDM in the current version (Fig. 4). Following export, the dataset is processed and validated against the LinkML schema, after which the CLI *phenopackets export* command generates the corresponding Phenopackets (Fig. 3b). A free-of-charge Bioportal API key is required to retrieve ontology labels during this process. The export engine leverages RareLink’s DataProcessor class to convert REDCap codes into valid *OntologyClass* elements within the Phenopacket structure. Mapping logic is defined in the BaseMapper class to support the necessary Phenopacket blocks. By integrating mapping, creation, writing, and validation functionalities within a single pipeline, the engine streamlines the export process. While the mappings are preconfigured for the RareLink-CDM, they remain extensible. Developers seeking to adapt the Phenopacket engine for other data models should follow RareLink’s guidelines for building ontology-based REDCap instruments. Detailed setup instructions for extending the engine are provided in the Phenopackets Module section of the documentation^17^.

### Clinical usability

Evaluation of the RareLink framework demonstrated usability and interoperability across diverse REDCap environments. During testing, users highlighted the intuitive documentation, the user-friendly CLI, and automated export pipelines as key strengths. The system successfully supported the export and analysis of all elements defined in the ontology-based RD-CDM that were mapped to either FHIR or Phenopackets, with the sole exception of the family history section in Phenopackets^14^, demonstrating its adaptability and coverage. Notably, predefining cohort-specific elements and customising the RareLink-CDM improved usability and consistency, facilitating subsequent registry deployment and data analysis. Its integration into the adopted REDCap infrastructure was noted as a major advantage, offering a scalable solution as demonstrated in phase two and three. However, REDCap’s typical separation from hospital information systems was identified as a barrier to seamless integration with clinical workflows. Conversely, utilising the RareLink-CDM’s LinkML schema and the RareLink-Phenopacket pipeline independently of REDCap increased efficiency in generating Phenopackets for retrospective cohorts. While the framework’s modular design and extensive guidance support its reusability across various research settings, the evaluation also highlighted challenges for users without a background in coding or interoperability. Users unfamiliar with command-line tools required additional support to operate the framework effectively. Effective clinical usability depended on precise local semantic annotation, with domain experts engaged to ensure that the chosen ontology terms reflected site-specific meaning and supported consistent data interpretation. To support these issues, feedback mechanisms embedded in the documentation and GitHub repository^17^ are actively collecting input for future enhancements.

Deployment teams in South Africa and Japan provided formative feedback, reinforcing that semantic alignment was a prerequisite for meaningful clinical use, and highlighting the framework’s scalability, the value of predefined cohort elements for harmonisation, and the need for tailored onboarding for non-technical users. To date, we have not conducted a combined international cohort analysis, but feedback from deployments is shaping the design and harmonisation of potential cross-site studies. We also note that using RareLink in different settings can introduce bias through mapping choices, local capture heterogeneity, and resolution fallbacks when ontology terms cannot be matched exactly. These risks were addressed through expert-reviewed semantic alignment, iterative refinement based on site feedback, and consistency checks with simulated data.

### Canadian Inborn Errors of Immunity National Registry

The RareLink framework demonstrated both scalability and extensibility through its application in the Canadian Inborn Errors of Immunity National Registry (CIEINR)^20^. The collaboration with CIEINR served as a key use case for evaluating extensibility and scalability. In this domain-specific implementation, the core RareLink-CDM was extended with additional clinical fields tailored to immunodeficiency-specific needs. Drawing from this experience, we developed guidelines for creating REDCap instruments compatible with the RareLink framework and provided detailed documentation on adapting the modular Phenopacket module for export. Iterative trial data capture, continuous feedback loops, and refinement processes informed these developments. Evaluation showed that the core RareLink-CDM sufficiently covered key data elements, such as demographics, patient clinical status, and genetic information. Additional domain-specific sections, such as phenotypic features, were incorporated through modular extensions and controlled value sets. Mandatory elements were preserved to maintain interoperability, and rule-based validation ensured consistency across the extended model. The CIEINR is in the implementation stage and has been proven operational for Phenopackets in the piloting phase. Specific development and analysis on the use of the RareLink framework will be reported elsewhere. This use case confirmed that RareLink can be adapted for diverse clinical domains while preserving semantic and syntactic integrity.

### Evaluation cohort

The evaluation cohort comprised ten simulated individuals with Kabuki Syndrome type 1. For each case, data included basic demographics, phenotypic features, genetic findings and selected clinical measurements. Following data entry, records were exported to both FHIR instances and Phenopackets (Fig. 3b). The resulting JSON files are publicly available via our GitHub repository^17^. As illustrated in Supplementary Fig. 2, the exported data maintain consistent semantic representations across formats for each individual, while ensuring syntactic interoperability with the respective data standard

## Discussion

In this study, we present RareLink, an open-source framework built around REDCap that enhances interoperability for rare disease research, studies, and registries. Our framework integrates the ontology-based RD-CDM^14^ into REDCap, facilitating its usage across countries and use-cases. RareLink provides predefined REDCap instruments and extension guides that enable direct export to Phenopackets and FHIR resource instances. In addition, it supports both guided manual data capture and, via LinkML, semi-automated import of retrospective cohorts. To support usability and global uptake, we developed a centralised, openly accessible documentation online and a command-line interface for seamless interaction with local REDCap instances.

RareLink can notably enhance data precision and availability for large cohorts and international registries, while also supporting downstream and advanced analyses. Recent studies employing large cohorts have significantly advanced our understanding of rare diseases while highlighting the need for precise encoding and interoperability^21–23^. The European Reference Networks (ERNs)^24^ and the European Health Data Space^25^ hold promise for harmonising extensive rare disease data. Yet, many disease-specific registries contain highly specialised data incompatible with FHIR or Phenopackets. RareLink can seamlessly connect the ERDRI-CDS^26^ to these data standards through a ready-to-use implementation. Its FHIR profiles inherit those of the HL7 IPS and Genomics Reporting and could therefore be beneficial for global rare disease efforts^27^. The integration with Phenopackets facilitates the use of reusable analysis pipelines, such as the novel GPSEA (genotype-phenotype: statistical evaluation of associations)^28^ or PhEval (Phenotypic inference Evaluation framework)^29^.

RareLink does not currently provide a direct integration with the Observational Medical Outcome Partnership (OMOP) Common Data Model^30,31^. The underlying ontology-based RD-CDM was developed to harmonise ERDRI-CDS, HL7 FHIR, and GA4GH Phenopackets while accommodating rare-disease-specific ontologies and semantics^14^ that are not natively supported in OMOP^31^. Notably, FHIR natively relies on JSON for structured, machine-readable healthcare data exchange^32^, while SNOMED CT can leverage RDF (Resource Description Framework) to enable formal, semantically precise representation of ontology content^33,34^. This combination of structural standardisation (JSON) and semantic modeling (RDF) ensures both syntactic and semantic consistency at a level of precision critical for research-grade health data^6,35^. Although existing conversion tools^13,36,37^ allow mapping to OMOP as an intermediary step, a representation purely in OMOP typically cannot retain the full semantic and structural precision required by researchers—particularly when working with rare-disease-specific ontologies and complex phenotypic descriptions^7,30,31^.

RareLink embodies several core aspects of FAIR data stewardship by operationalising shared structural and semantic standards (FHIR, Phenopackets, and ontology-based data modeling), thereby improving findability, interoperability, and reusability of rare disease data across sites. Its integration of the ontology-based RD-CDM with export to data standards and the use of common ontologies can make data more discoverable and semantically consistent for downstream analysis. The readiness for distributed use aligns with FAIR’s emphasis on data prepared at source^10^. However, the framework is not yet fully FAIR in practice: coordination with higher-level harmonisation initiatives (e.g., HL7 and the European Rare Disease Registry Infrastructure) is still evolving, and distributed governance, provenance capture, and broader integration into federated networks remain works in progress. These gaps can limit seamless cross-institutional reuse and require continued alignment and harmonised metadata practices to move toward comprehensive FAIR compliance^10,38^.

Extending the RareLink-CDM model with the CIEINR^20^ provided valuable insights into establishing domain-specific common data elements^39^ that are compatible with our framework. The use of ontologies has been essential for preserving medical semantics while ensuring compatibility with the given data standards and other domain-specific organisations^8^. Although RareLink has proven effective for multi-site research due to its lightweight implementation around any REDCap instance, its success will ultimately rely on organisational interoperability and governance within each use case.

Important limitations to highlight include inherent constraints of REDCap. It is typically not integrated within clinical workflows or the electronic health records (EHR)^16^, which can necessitate additional resources and limit the effectiveness of a common data model^38^. A local RareLink installation can import FHIR data more easily via the Clinical Data Interoperability Service module^16^ using the RareLink-CDM profiles. Nonetheless, linking existing patient records within a cohort remains challenging due to heterogeneous underlying information systems, regulatory constraints, and the availability of required data elements in FHIR^16^. Further, native interoperability with semantic and syntactic technologies such as Semantic Science Integrated Ontology (SIO)^40^ is currently not provided in RareLink. However, Semantic Web Technologies can, for instance, be used to describe and interlink ontology within SNOMED CT using RDF^33,34,41^. Further, LinkML supports JSON-LD^42^ (as described in its documentation), enabling the RareLink-CDM to be serialised and consumed in JSON-LD format^43^.

Requiring a country-specific SNOMED CT license for RareLink and its underlying RD-CDM^14^ creates a substantial barrier in non-member countries, as there is currently no equivalent replacement for the elements encoded in SNOMED CT, though future versions may explore alternative or complementary solutions. Additional licensing issues with REDCap or usage constraints may arise from installing external software in clinical environments and from the requirement for BioPortal connectivity, as some sites may be reluctant to connect clinical systems to external web services. Further, some ontologies in BioPortal are not actively maintained or reliably resolvable, and some may lack the clinical curation needed. Although the RareLink-CDM defines the required ontologies within its REDCap instruments and LinkML schema, local expert review may be required to resolve potential issues related to ontologies in BioPortal. Similarly, when users employ the semi-automated import of retrospective data and manually specified ontology versions, discrepancies can emerge between the live ontology releases and the versions encoded in their source data, necessitating careful review and validation. Another current limitation is that RareLink and its underlying model^14^ rely on English-language ontology labels from both BioPortal and the model itself for term resolution and presentation. Full multilingual support (e.g., alternate-language synonyms or localised mappings) is not yet implemented, which may reduce usability and semantic accuracy in non-English-speaking deployments, although this could be partially mitigated using REDCap’s Multi-Language Management^44^.

Another limitation relates to the novelty of the data model^14^ implemented into RareLink. While it incorporates the ERDRI-CDS^26^ and links to HL7 FHIR and the GA4GH Phenopacket Schema, to our knowledge, this study represents its first implementation with real world data. Consequently, even though a direct connection with FHIR and Phenopackets is ensured, the introduction of another common data model may contribute to further fragmentation and bias among cohorts^38^. Moreover, the RareLink-CDM FHIR profiles and Implementation Guide remain in draft status and subject to formal governance—including versioning, conformance testing, stakeholder review, and the incorporation of changes arising from the ballot process—before achieving formal approval. Although the ontology-based RD-CDM derived inspiration from multiple rare disease data models^14,30,33,45^ including the CARE-SM^46^, semantic interoperability remains immature at this stage. However, the 16 core elements of the ERDRI-CDS provide a minimal common foundation between the RareLink-CDM and these models^14,30,33,45,46^. Thus, the RareLink Implementation Guide can provide a practical foundation for linking these frameworks to formalise common FHIR definitions of their shared elements. These RareLink-FHIR profiles, aligned with the IPS and Genomics Reporting profiles, can also help position the ERNs^24^ for seamless integration with GA4GH Phenopackets or broader infrastructures such as the European Health Data Space^47^. Our method of standardising the extensional, domain-specific CIEINR model to enable interoperability with FHIR and Phenopackets can provide a template that could be adopted for other European domain-specific common data elements^39^. Finally, country-specific requirements, multilingual implementation challenges, and interoperability limitations highlight the need for coordinated alignment with international stakeholders, such as HL7, the IPS, and the GA4GH^38^, and existing international rare disease initiatives, including the European Joint Programme on Rare Diseases, the European Rare Diseases Research Alliance^48^, and the ERNs^24^.

Future research should also leverage advanced FHIR infrastructures and features, such as SMART-on-FHIR^49^ and FHIR Questionnaires^50^ to enhance integration with EHRs and apply RareLink’s specifications to other clinical information systems. This would position Phenopackets closer to routine clinical data, thereby increasing available data for precision medicine and federated analyses^7,35,51^. Moreover, broader implementation of RareLink across additional use cases, cohorts, and countries is warranted. Ultimately, extensive feedback will be essential to refine the documentation, engine, command-line interface, and specifications, thus improving adaptability and overall utility.

In conclusion, RareLink enhances data precision for rare disease experts by leveraging the widely adopted REDCap system. Its preconfigured pipelines and international accessibility make it a valuable resource for researchers. By enabling standardised analysis algorithms^28,29^ and linking modern registries^24^, RareLink can significantly advance our understanding and treatment of rare diseases. Its effectiveness, however, centres on the community-driven and open-source effort. Only through such collaborative engagement, RareLink can evolve into a reliable global resource for rare disease research in REDCap, supporting equity regardless of financial or geographic constraints.

## Methods

### Overall project design

**Fig. 5:**
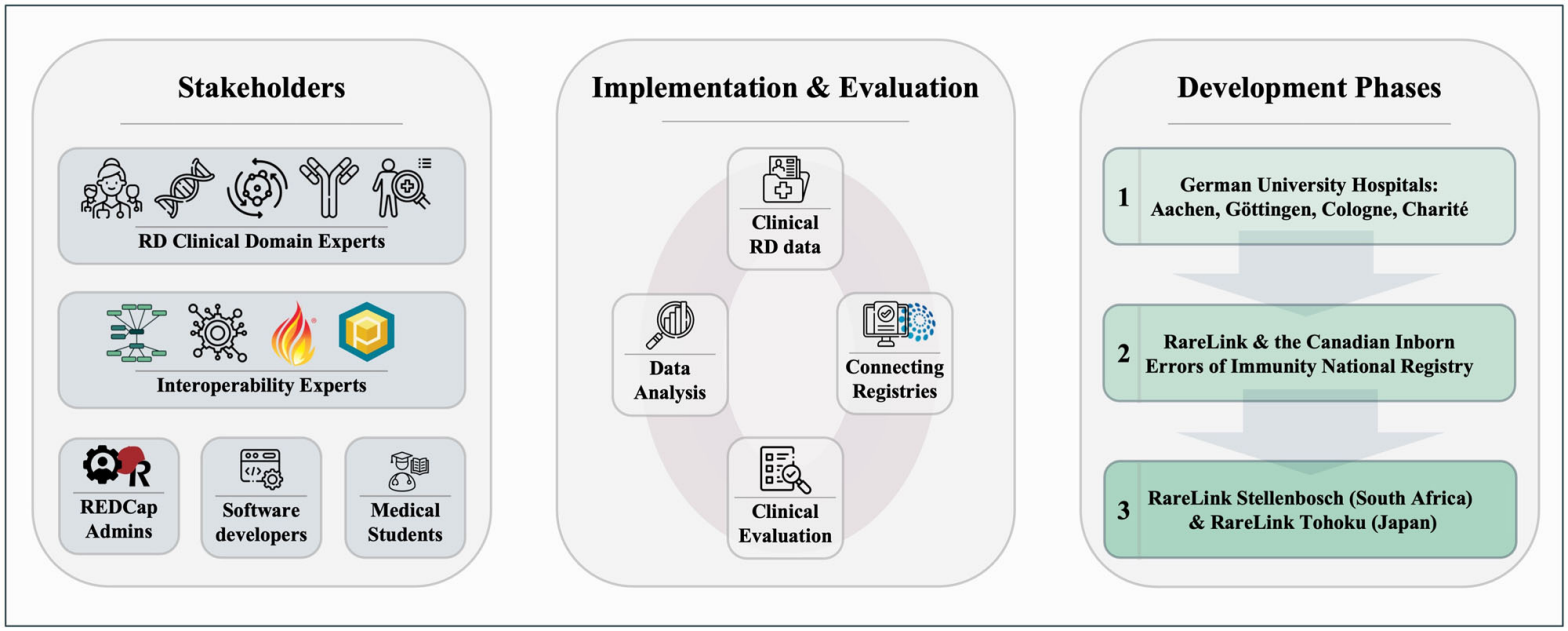
Development of the RareLink framework. This figure illustrates the methodological foundation of the RareLink framework, including key stakeholders, implementation, evaluation, and three development phases. Implementation involved clinical rare disease data from diverse sources further specified in the main text; applied for data analysis and registry linkage, including the European Reference Networks. Development proceeded through three overlapping phases: (1) deployment at German university hospitals in Aachen, Göttingen, Cologne, and Charité Berlin; (2) integration with the Canadian Inborn Errors of Immunity National Registry, and (3) implementation at Stellenbosch University (South Africa) and Tohoku University (Japan). RD rare diseases.

Our work on RareLink followed an iterative and collaborative development process over the past three years (Fig. 5). A diverse group of stakeholders contributed to its development and validation, providing domain-specific expertise at each stage. Clinical experts from metabolic, neuromuscular, immunodeficiency, clinical genetics, and other rare diseases guided the clinical applicability. Interoperability experts supported the implementation of ontologies and health data standards. Software developers and REDCap administrators contributed to the technical development of the framework, while doctoral and medical students assisted with data capture, analysis, and iterative prototyping.

### Technical development

Developed with Python 3.12 and an object-oriented design, the RareLink codebase is available on GitHub under an open-source Apache v2.0 license and on the Python Package Index (PyPI)^17^. We have built several RareLink Python packages, including utilities, the Typer-based command line interface, and a RareLink-Phenopacket module. Additionally, a Sphinx-based RareLink documentation, linked to our GitHub repository, centralises all information required for the installation, usage, and implementation of the RareLink framework^17^.

We embedded the entire ontology-based RD-CDM^14^ into REDCap, with each section represented as one instrument. All ontologies associated with the data elements and value sets were incorporated in REDCap’s variable name and choices fields with their respective Internationalized Resource Identifiers prefixes while adhering with its naming constraints (i.e. lowercase letters, underscores only, and no additional special characters). Within REDCap’s Field Annotations, each element was defined along with its semantic codes, their ontology versions, and mappings to FHIR and Phenopackets, creating an integrated expression repository.

By leveraging REDCap’s repeating instruments and required fields, we defined the cardinalities for our embedded data model to ensure compliance with the HL7 International Patient Summary (IPS) v2.0.0^52^, the HL7 Genomics Reporting v3.0.0^53^, and the GA4GH Phenopacket Schema v2.0^7^. The resulting REDCap data model and accompanying data dictionary, named *RareLink-CDM* (short for RareLink Common Data Model), was defined with LinkML^15^ v1.8.7, a unified data modeling language with integrated Python functionalities. We use this specification to process and validate exported REDCap records and leverage the LinkML-Map module to support semi-automated import of tabular data into REDCap. Detailed specifications of the RareLink-CDM are provided in the corresponding section of the RareLink documentation^17^.

The ontology versions for all concepts in the RareLink-CDM are automatically synchronised from the RD-CDM repository through its PyPI package^18^, which in turn tracks and incorporates the most current BioPortal ontology releases. These versions are automatically embedded into the RareLink-CDM REDCap data dictionary, its corresponding LinkML schema, and the FHIR/Phenopacket pipelines to prevent discrepancies between BioPortal search fields in REDCap, BioPortal label fetching in the Phenopackets pipeline, and the model’s own data elements and value sets. For retrospective data capture, ontology versions require careful validation against the current RD-CDM version to ensure consistency between the chosen version (e.g., for the Disease field [5.1] or the Phenotypic Feature field [6.2.1]) and the version defined by the model for other elements.

Mapping and export of our RareLink-CDM to HL7 FHIR were performed using the open-source toFHIR engine^19^. We uploaded the FHIR v4.0.1 Base Resources, the Genomics Reporting v3.0.0, and IPS v2.0.0 profiles into the aforementioned engine, ensuring that exports of the RareLink-CDM produce validated FHIR resource instances compliant with these resources and profiles. Additionally, we developed specific RareLink-CDM v2.0.0 profiles using the FHIR Shorthand (FSH) Language and the FSH compiler SUSHI Unshortens SHorthand Inputs (SUSHI) v3.15.0. A set of example instances were validated against the corresponding Structure Definitions and published using the IG Publisher v1.8.25.

The RareLink-Phenopacket module was designed to be compatible with other REDCap data models that are embedding ontologies. The Python package includes general mapping and creation functions for multiple Phenopacket blocks and Python classes for processing while integrating the Phenopackets Python package to ensure automatic validation. The mapping configuration for the RareLink-CDM was defined according to the underlying RD-CDM^14^. Further, we developed guidelines for defining other REDCap data models and their corresponding configurations that can also utilise the module^17^.

### Implementation and clinical applications

We deployed RareLink in multiple real-world settings to support both registry development and data analysis, with iterative refinements to enhance international scalability and semantic consistency. Implementations encompassed a range of rare diseases, including rare neuromuscular, neurometabolic, hereditary bone, and developmental disorders, inborn errors of immunity, and undiagnosed cases, using both retrospective and prospective clinical data extracted from the electronic health records (EHR) and local information systems.

This study was conducted in compliance with all relevant ethical regulations and the principles of the Declaration of Helsinki. Deployments were conducted in three overlapping phases, some of which are still ongoing. In phase one, RareLink was implemented at the four German university hospitals Berlin, Cologne, Aachen, and Göttingen. Phenopacket-based analyses were performed, and data were captured for local registries and the European Reference Networks. Ethical approval was obtained from Berlin (approval number: EA2/242/22) and Cologne (approval number: 21-1477_1). In Cologne data was collected as part of the European Reference Network on rare bone diseases (ERN BOND). In Aachen, data use was governed by institutional registry protocols under the responsibility of the respective clinical data custodians. In Göttingen data was captured as part of the EURO-NMD (Neuromuscular disorder) Registry^54^ (approval number: 3/2/25Ü). In phase two, we integrated RareLink with the Canadian Inborn Errors of Immunity National Registry (CIEINR)^20^, whose extended data model informed the design of additional domain-specific forms compatible with our framework with ethical approval numbers from the Izaak Walton Killam Hospital for Children in Halifax (IWK-REB:1028743) and the University of Calgary (UofC-REB:23-0108).

In phase three, RareLink is currently being implemented at Stellenbosch University (South Africa) and Tohoku University (Japan), with initial testing conducted using synthetic data and preparations underway for real-world clinical deployment within the Undiagnosed Disease Programme in South Africa^55^ (ethical approval number from Stellenbosch University Health Research Ethics Committee: N18/03/031) and partners in Japanese rare disease projects, such as the Initiative on Rare and Undiagnosed Diseases at Keio University Hospital^56^. While this study does not report detailed cohort-level findings, its focus was on evaluating global scalability, adapting to country-specific and international requirements, and refining the framework accordingly.

Feedback was continuously gathered throughout the iterative deployment process via informal exchanges, implementation meetings, and hands-on user testing. Rather than following a formalised evaluation protocol, feedback was integrated through a collaborative, back-and-forth process with clinical and technical partners. As such, the evaluation reflects a qualitative synthesis of observations and implementation experiences across diverse settings. Terminology alignment sessions with clinical and registry partners were conducted, the resulting mappings were externally reviewed to ensure local semantic alignment. Identified discrepancies were incorporated through iterative refinement, and for specific ontologies formal change requests were submitted when needed. To assess the semantic precision of the generated data, we created a simulated cohort of ten individuals with Kabuki syndrome type 1 based on published cases and entered their data using our RareLink-CDM. In addition to genetic findings, data on the disease, phenotypic features, clinical measurements, and demographics were captured. We exported these records to both FHIR and Phenopacket formats and compared the output for semantic consistency (Supplementary Fig. 2). The complete evaluation cohort including its REDCap source data, Phenopackets, and FHIR instances are publicly available in our GitHub repository^17^.

## Supplementary information

Supplementary Material.

## Data Availability

The dataset of the simulated evaluation Kabuki cohort as REDCap data, Phenopackets, and FHIR instances are available in the RareLink repository, (https://github.com/BIH-CEI/rarelink/tree/develop/res/evaluation_cohort).

## Code availability

The underlying code for this study is available in the RareLink GitHub repository and can be accessed via this link https://github.com/BIH-CEI/RareLink. The documentation is accessible at https://rarelink.readthedocs.io/en/latest/index.html and the RareLink FHIR implementation guide and the corresponding RareLink-CDM FHIR profiles are available at https://bih-cei.github.io/rarelink/.
