# Supplementary material for: RareLink: scalable REDCap-based framework for rare disease interoperability linking international registries to FHIR and Phenopackets
Source: NPJ Genom Med. 2025 Nov 18;10:72. doi: 10.1038/s41525-025-00534-z (PMC12627670; doi:10.1038/s41525-025-00534-z)
Supplement: Supplementary file 1 — Supplementary Material. [file 41525_2025_534_MOESM1_ESM.pdf]

Supplementary Material

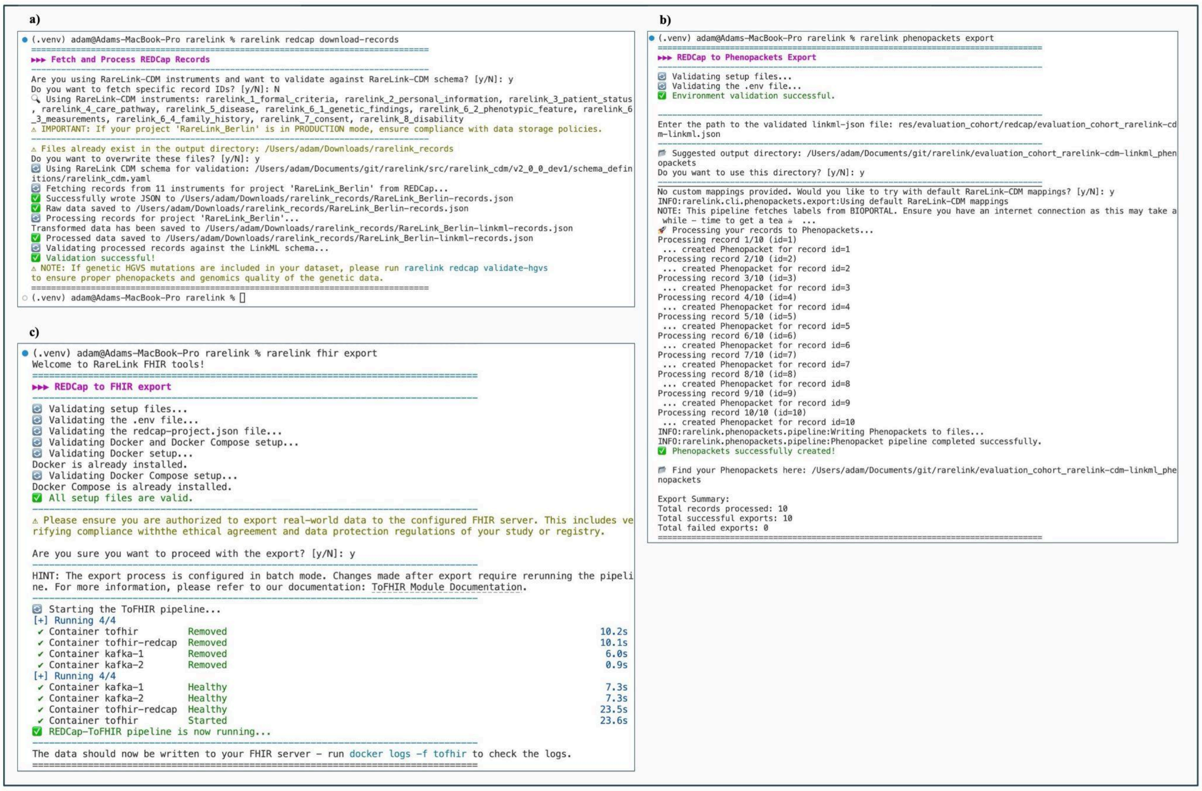

Supplementary Fig. 1 | Complete console output illustrating the data-processing workflow for the evaluation cohort. a) The *rarelink redcap download-records* command retrieves all records from the local REDCap instance and validates them against the RareLink-CDM LinkML schema. b) The *rarelink phenopackets export* command transforms validated LinkML data into GA4GH Phenopackets, resolving ontology labels via BioPortal. c) The *rarelink fhir export* command invokes the toFHIR engine in Docker to convert REDCap data directly into FHIR R4 resources. CDM=common data model. CLI=command-line interface. FHIR=Fast Healthcare Interoperability Resources. LinkML=Linked Data Modeling Language.

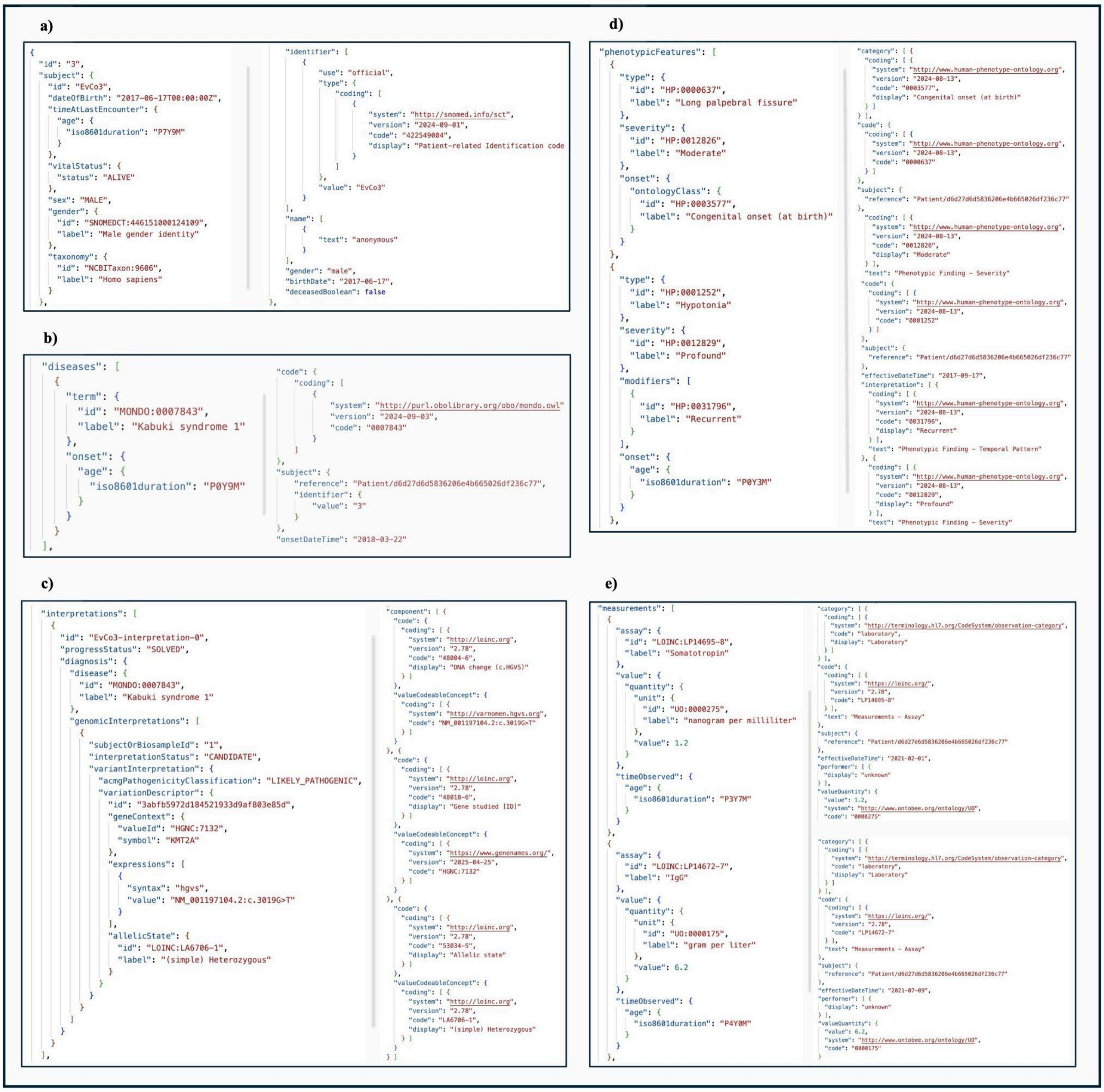

Supplementary Fig. 2 | Representative JSON excerpts from a Phenopacket (left part in each subfigure) and corresponding FHIR resources (right part in each subfigure) for a single individual enrolled in the Kabuki Syndrome 1 evaluation cohort illustrating patient demographics, disease diagnosis, genetic findings, phenotypic features, and clinical laboratory measurements. a) The individual is a male (REDCap record ID: 3; cohort-specific ID: EvCo3), born on 2017-06-17 and at the last encounter alive. b) The diagnosis of Kabuki Syndrome 1 was made with an onset date of 2018-03-22, corresponding to an age of 9 months. c) Genetic analysis identified a likely pathogenic, heterozygous variant in the KMT2A gene associated with the diagnosis. d) Phenotypic presentation included a moderately expressed long palpebral fissure with congenital onset and recurrent profound hypotonia manifesting at 3 months of age. e) A somatotropin concentration of 1.2 ng/mL was recorded on 2021-02-01, when the individual was 3 years and 7 months old, and an IgG concentration of 6.2 g/l was recorded on 2021-07-09, when the individual was 11 years and 4 months old. The complete set of synthetic test data used in this evaluation cohort is available in our public GitHub repository (https://github.com/BIH-CEI/rarelink/tree/develop/res/evaluation_cohort).
